# Increased monocyte distribution width in COVID‐19 and sepsis arises from a complex interplay of altered monocyte cellular size and subset frequency

**DOI:** 10.1111/ijlh.13941

**Published:** 2022-08-01

**Authors:** Martina Cusinato, Linda Hadcocks, Simon Yona, Timothy Planche, Derek Macallan

**Affiliations:** ^1^ Institute for Infection and Immunity St. George's, University of London London UK; ^2^ Infection Clinical Academic Group St. George's University Hospitals NHS Foundation Trust London UK; ^3^ The Institute of Oral and Biomedical Research, Faculty of Dental Medicine Hebrew University, Ein‐Kerem Campus Jerusalem Israel; ^4^ Infection Care Group St. George's University Hospitals NHS Foundation Trust London UK

**Keywords:** COVID‐19, flow cytometry, MDW, monocytes, sepsis

## Abstract

**Introduction:**

Monocyte distribution width (MDW), a parameter generated alongside full blood counts (FBC) in new‐generation haematology analysers, has been proposed as a diagnostic test for severe infection/sepsis. It represents the standard deviation (SD) of the monocyte mean volume (MMV).

**Methods:**

This study aimed to compare monocyte volumetric parameters retrieved by the UniCel DxH 900 haematology analyser (MMV and MDW) against corresponding parameters from the same sample measured using flow cytometry (forward scatter [FSC] mean and SD) in combination with phenotypic characterization of monocyte subtypes. We analysed blood samples from healthy individuals (*n* = 11) and patients with conditions associated with elevated MDW: sepsis (*n* = 26) and COVID‐19 (*n* = 15).

**Results:**

Between‐instrument comparisons of monocyte volume parameters (MMV vs. FSC‐mean) showed relatively good levels of correlation, but comparisons across volume variability parameters (MDW vs. FSC‐SD) were poor. Stratification on sample type revealed this lack of correlation only within the sepsis group. Flow cytometry analysis revealed that in healthy controls intermediate monocytes are the largest and non‐classical the smallest cells. In each disease state, however, each monocyte subset undergoes different changes in volume and frequency that together determine the overall configuration of the monocyte population. Increased MDW was associated with reduced classical monocyte frequency and increased intermediate monocyte size. In COVID‐19, the range of monocyte sizes (smallest to largest) reduced, whereas in sepsis it increased.

**Conclusion:**

Increased MDW in COVID‐19 and sepsis has no single flow cytometric phenotypic correlate. It represents—within a single value—the delicate equipoise between monocyte subset frequency and size.

## 1 INTRODUCTION

Monocyte distribution width (MDW) is a parameter generated alongside full blood counts (FBC) in new generation haematology analysers. The value of MDW as a diagnostic tool has been investigated in sepsis and coronavirus disease (COVID‐19).^1^, ^2^, ^3^, ^4^, ^5^, ^6^, ^7^, ^8^, ^9^, ^10^, ^11^ Increased MDW values are associated with both disorders; however, the mechanistic processes that underpin these observations remain less clearly defined.

MDW is produced by the haematology analyser UniCel DxH 900 Coulter Cellular Analysis System (Beckman Coulter, Miami, Florida, USA), which uses (i) the Coulter principle of automated cell counting and sizing and (ii) the volume, conductivity, and multiple angles of light scatter (VSC) analysis. This technology identifies monocytes in the volume (V) versus rotated light scatter (RLSn) data plot, where MMV is the distribution centre of the monocyte cell volume and MDW its standard deviation (SD).^12^, ^13^

Although MDW is a parameter which treats monocytes as a single population; the cells included in the “monocyte” gate, however, can better be considered as a cluster of morphologically and phenotypically distinct subpopulations. Three main subsets are conventionally recognized: classical (CD14^+^CD16^−^), intermediate (CD14^+^CD16^+^) and non‐classical (CD14^lo^CD16^+^) monocytes.^14^, ^15^ The blood monocyte pool is highly dynamic population, particularly during system inflammation. The population dynamics and consequently the composition of the monocyte subsets from the time of “injury” coupled with a complex developmental and kinetic trajectory of each subpopulation may facilitate this population of cells as a temporal diagnostic marker.^14^, ^16^ Flow cytometry can identify discrete monocyte subpopulations, but it is not routinely used in clinical practice, unlike the FBC which is perhaps the single most common investigation performed in medical patients.^17^ Understanding the cell attributes which underlie both measurements will optimize the yield of information from FBC analysis.

This study aimed to compare monocyte volumetric parameters retrieved by the UniCel DxH 900 analyser (MMV and MDW) against the corresponding parameters measured using flow cytometry (forward scatter [FSC] mean and FSC‐SD). Although both instruments measure cell volume (mean and standard deviation), their methodology is incommensurate; so instead of determining a level of agreement, we explored graphical relationships and assessed whether their capacity to discriminate between healthy and diseased distributions was comparable. To investigate this, we analysed blood samples from patients with conditions known to elevate MDW values (sepsis and COVID‐19), comparing results to healthy controls expected to have normal MDW values.^2^ We analysed each sample in parallel on both instruments. The UniCel DxH 900 analyser is only able to produce two monocyte volumetric parameters per sample (MMV and MDW), but flow cytometry can provide FSC‐mean and FSC‐SD for all monocyte subtypes; so between‐instrument comparisons were done using measures corresponding to all monocytes, but the additional information on monocyte subpopulations obtained from flow cytometry was leveraged to inform the descriptive analysis.

## 2 METHODS

### 2.1 Specimens and ethics statements

This study used excess diagnostic blood samples taken from routine FBC of patients admitted to St. George's University Hospitals NHS Foundation Trust (SGHFT), a London University Hospital. Specimens were obtained from adult patients (aged ≥18 years) admitted (i) to the emergency department (ED) with a high clinical suspicion of sepsis, and (ii) with severe acute respiratory syndrome coronavirus‐2 (SARS‐CoV‐2) infection. Control specimens were obtained from healthy volunteers.

Sepsis participants were enrolled as part of an ongoing observational study (NCT04300530) approved by national Health Research Authority (HRA) and Health and Care Research Wales (HCRW) and Coventry and Warwickshire Research Ethics Committee (20/WM/0103). COVID‐19 participants were enrolled as part of DARTS study (NCT04351646), approved by national HRA and HCRW and Oxford Research Ethics Committee (20/SC/0171). Both ethical approvals allowed recruitment without formal written consent from participants since sampling utilized excess diagnostic material. Healthy volunteers gave written informed consent following protocols approved by London Central Research Ethics Committee (13/LO/1621). Study procedures complied with all relevant ethical regulations, following the principles of the Declaration of Helsinki (2008) and the International Conference on Harmonization (ICH) Good Clinical Practice (GCP) guidelines.

### 2.2 Participants

Participants were identified (consecutively) by treating physicians, aware of the relevant inclusion/exclusion criteria for the corresponding parent studies. The recruitment of sepsis and COVID‐19 participants took place between April and September 2020; and of healthy volunteers between January and March 2021. The inclusion/exclusion criteria were determined by the parent study and thus differed between groups, but all samples were handled in the same way (see Data S1).

Fifty‐nine participants were recruited: 28 sepsis, 20 COVID‐19 and 11 healthy controls. Five COVID‐19 samples were excluded due to missing MDW values, and two sepsis samples were excluded from the quantitative analysis due to aberrant staining patterns preventing accurate quantification of monocyte gates. Fifty‐two independent samples were thus included in the final analysis. Each sample was analysed in parallel on both the UniCel DxH 900 and by flow cytometry.

### 2.3 Processing samples

For each participant, whole human peripheral blood was collected routinely in sterile vacutainer tubes containing K2 EDTA (dipotassium ethylenediaminetetraacetic acid). Prior to sample processing we confirmed that cryopreservation did not introduce a bias or artefact (data not shown). Details on the procedures for processing blood samples and direct labelling are included in the Data S1.

### 2.4 Flow cytometry gating strategy

Data were acquired (between 500 000 and 800 000 events) using a CytoFLEX S flow cytometer (Beckman‐Coulter) and analysed using FlowJo software (FlowJo, LLC, version 10.6.2). Full details on optical configuration and flow cytometry settings, compensation matrix, details on staining antibodies, and panel description are included in Data S1.

**FIGURE 1:**
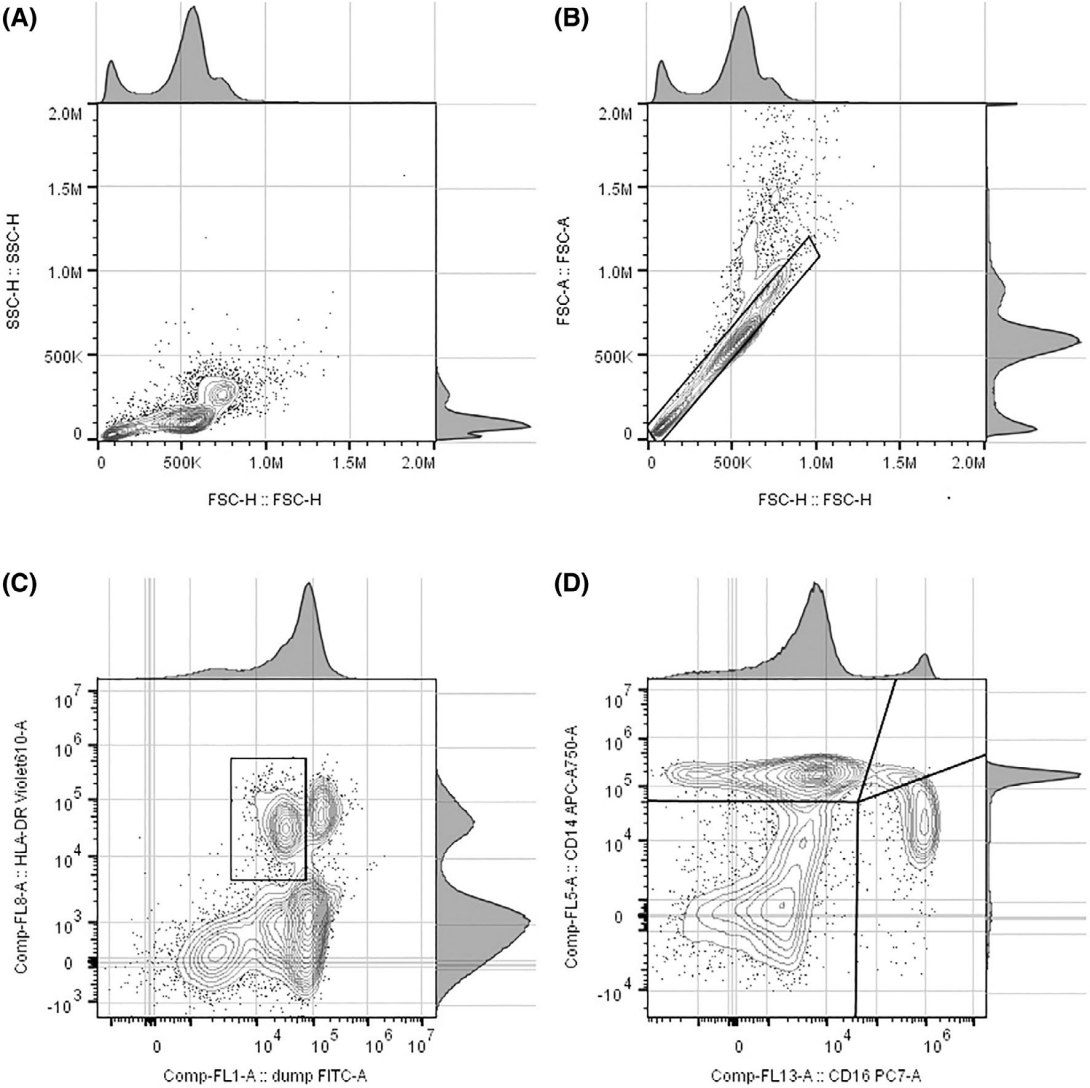
Flow cytometry (CytoFLEX) gating strategy. (A) Forward scatter height (FSC‐H) versus side‐scatter height (SSC‐H). (B) Forward scatter height (FSC‐H) versus forward scatter area (FSC‐A). (C) Fluorescence for HLA‐DR positive cells using fluorochrome BV605 versus fluorescence for lineage‐negative cells and dead cells using FITC. (D) Fluorescence for CD14 positive cells using fluorochrome APC‐Cy7 versus fluorescence for CD16 positive cells using fluorochrome PE‐Cy7

The gating strategy is shown in Figure 1. Briefly, monocytes were identified by forward (FSC‐H) and side‐scatter (SSC‐H) parameters, doublet‐exclusion, and positive selection for HLA‐DR^+^ with negative selection for CD3, CD19, CD20, CD56, CD66b, and dead cells. The resulting population, referred to as “HLA‐DR^+^ cells” was further separated into: (i) CD14^+^CD16^−^ classical monocytes; (ii) CD14^+^CD16^+^ intermediate monocytes; (iii) CD14^lo^CD16^+^ non‐classical monocytes, and (iv) CD14^lo^CD16^lo^ double‐negative cells. Subpopulation structure and function was defined by expression of CD192 (CCR2), CD45RA, CX_**3**_CR1 and CD169.^18^ A more detailed description of the gating strategy has been included in the Data S1.

### 2.5 Statistical analysis

The distribution of baseline characteristics available was assessed across the different groups analysed (controls, COVID‐19, and sepsis) using proportions and means or medians as appropriate according to the nature of the variable. Relationships between variables were explored graphically (by means of scatterplots, density and box plots) and the strength of the correlation for numerical variables was quantified using Pearson product–moment correlation (*r*). Statistical tests used are indicated in the appropriate table footer and/or figure legend, and .05 was used as a level of significance. Missing data was not imputed and were handled by exclusion.

Predictive modelling was used to explore the determinants of volume variability. Linear regression was used to model volume variability and the validity of assumptions was assessed using graphical and formal statistical methods (i.e. standard residuals vs. fitted, theoretical quantile vs. empirical quantile, scale‐location and residuals vs. leverage plots, Cook's distance and Shapiro–Wilk test), additionally the potential impact of influential points was explored through sensitivity analysis. Different sets of variables were considered for the model build to explore different assumptions: model A, considered all CytoFLEX monocyte parameters and used monocyte FSC‐SD as a response variable; model B considered the same parameters but used MDW as a response variable. Three extensions of model B were explored, one adding CytoFLEX parameters for double‐negative cells (model B1), the second, fitting sample groups as a multiplicative parameter (model B2), and the third, adding UniCel DxH 900 monocyte count and MMV to the initial set of CytoFLEX monocyte parameters (model B3). All continuous variables were fitted as linear parameters to allow for a more parsimonious model and none of the parameters were forced in. A backward–forward stepwise deletion strategy was used with Bayesian Information Criterion (BIC) as a condition for removal.^19^, ^20^ Interactions were assessed parsimoniously in the final model and compared to the main‐effect model using likelihood ratio test.

This was a descriptive analysis without a formal sample size calculation (or predetermined analysis plan). Data management and statistical analyses were carried out using R (R Core Team, version 3.6.3, Vienna, Austria. Packages: tidyverse, broom, lubridate, MASS). Graphs and plots were created using both R (ggplot2) and FlowJo (FlowJo LLC, version 10.6.2).^21^, ^22^, ^23^, ^24^, ^25^

## 3 RESULTS

### 3.1 Study population and monocyte volumetric parameters

This analysis included 52 independent samples from patients with sepsis (*n* = 26), COVID‐19 (*n* = 15), and healthy volunteers (*n* = 11). The median age in controls was 46.0 years (IQR 30.5, 57.0), in COVID‐19 patients 62.0 years (IQR 46.5, 72.0), and in sepsis 71.5 years (IQR 56.5, 80.5). The proportion of males was largest in the sepsis group (80.8%, 21 of 26) compared to 66.7% (10 of 15) in COVID‐19 and 36.4% (4 of 11) in controls.

Table 1 shows the distribution of FBC and monocyte volumetric parameters across the different groups. Among sepsis patients, source of infection was predominately respiratory in 9/26 (34.6%), unknown in 7/26 (26.9%), urogenital in 4/26 (15.4%), abdominal in 3/26 (11.5%), ENT or soft tissue in 2/26 (7.8%) and missing in 1/26. Median sequential organ failure assessment (SOFA) scores at ED admission were 2.0 (IQR 2.0, 4.0), and only 2/26 (7.6%) sepsis patients had a positive blood culture result within 15 days of admission. All COVID‐19 patients had SARS‐CoV‐2 PCR‐confirmed infection. In terms of frailty, 3/15 (20.0%) had a frailty score <2 (fit or very fit), 8/15 (53.3%) scored 3 (managing well), 3/15 (20.0%) scored 4 (vulnerable), and 1/15 patient (6.7%) scored >7 (severely frail). Among COVID‐19 patients, 10/15 (66.7%) were admitted to the intensive care unit (ICU).

**TABLE 1:** Distribution FBC and monocyte volumetric parameters across groups

	Controls (*n* = 11)	COVID (*n* = 15)	Sepsis (*n* = 26)	*p* value
**WCC**				
Mean (SD)	5.8 (.8)	11.4 (4.2)	13.6 (8.8)	*p* ^1^ < .001
Med. (IQR)	5.9 (5.2, 6.4)	10.4 (8.0, 13.6)	11.3 (8.4, 15.9)	*p* ^2^ = .745
Missing	2	0	0	
**Neu**.				
Mean (SD)	3.3 (.6)	8.8 (4.0)	11.7 (8.3)	*p* ^1^ < .001
Med.(IQR)	3.5 (2.6, 3.7)	8.0 (5.4, 10.9)	10.0 (6.1, 13.5)	*p* ^2^ = .330
Missing	2	0	0	
**Lym**.				
Mean (SD)	1.9 (.5)	1.6 (.8)	1.0 (.5)	*p* ^1^ < .001
Med. (IQR)	1.7 (1.7, 2.1)	1.5 (1.0, 2.1)	1.0 (.5, 1.2)	*p* ^2^ = .012
Missing	2	0	0	
**Monocytes**				
Mean (SD)	.4 (.1)	.9 (.4)	.9 (.6)	*p* ^1^ < .001
Med. (IQR)	.4 (.3, .4)	.8 (.6, 1.1)	.6 (.5, 1.2)	*p* ^2^ = .734
Missing	0	0	0	
**MMV**				
Mean (SD)	164.9 (5.0)	178.6 (11.7)	184.0 (14.1)	*p* ^1^ < .001
Med. (IQR)	163.0 (162.0, 167.0)	175.0 (171.0, 181.5)	181.0 (173.0, 190.5)	*p* ^2^ = .171
Missing	2	0	0	
**FSC‐mean**				
Mean (SD)	87.7 (3.8)	90.0 (2.6)	89.1 (5.1)	*p* ^1^ = .145
Med. (IQR)	87.8 (86.2, 88.0)	90.3 (88.6, 91.3)	89.4 (85.7, 92.5)	*p* ^2^ = .715
**MDW**				
Mean (SD)	16.0 (1.2)	23.0 (4.3)	26.5 (6.4)	*p* ^1^ < .001
Med. (IQR)	16.2 (15.3, 16.3)	23.8 (20.0, 24.8)	24.4 (21.3, 30.9)	*p* ^2^ = .130
**FSC‐SD**				
Mean (SD)	9.1 (.9)	8.8 (.6)	9.7 (1.2)	*p* ^1^ = .021
Med. (IQR)	9.1 (8.4, 9.8)	8.7 (8.4, 9.1)	9.5 (8.8, 10.5)	*p* ^2^ = .006

*Note*: The table shows cell counts in 109 cells/L. UniCel DxH 900 parameters (MMV and MDW) in units and CytoFLEX parameters (FSC‐mean and FSC‐SD) per 10 000 Units. *p*‐values correspond to the Kruskal‐Wallis test. *p* ^1^ corresponds to the test assessing differences across all groups. *p* ^2^ corresponds to the test comparing COVID‐19 versus sepsis.

Abbreviations: IQR, interquartile range; Lym, lymphocytes; Med, median; Neu, neutrophils; SD, Standard deviation; WCC, white cell counts.

**FIGURE 2:**
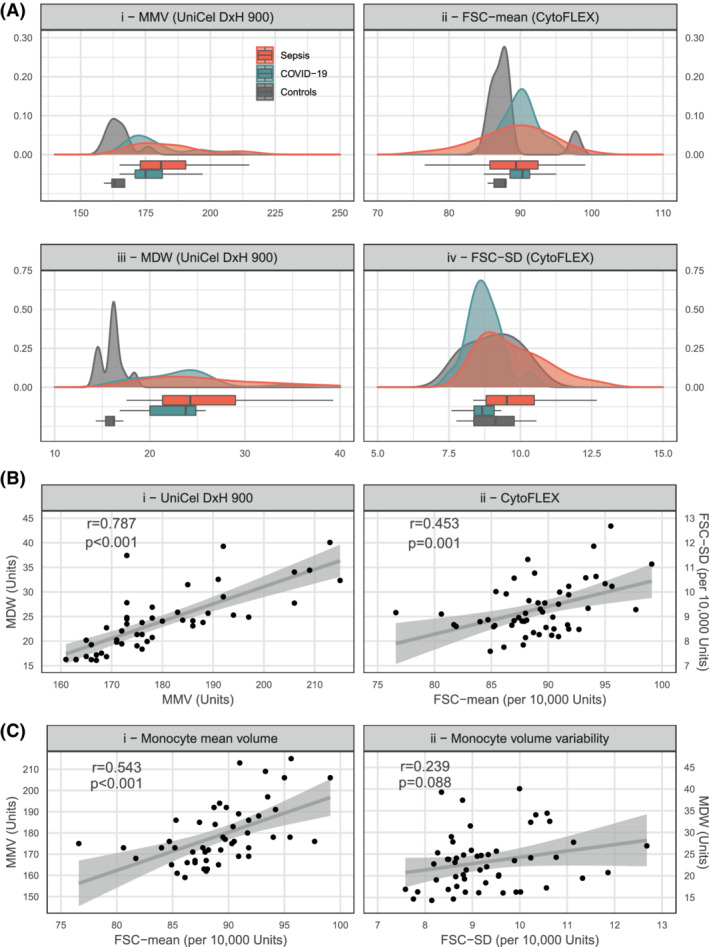
(A) Density plots and boxplots displaying the distribution of the mean monocyte volume (MMV and FSC‐mean) and monocyte volume variability (MDW and FSC‐SD) across instruments and for each group (controls, COVID‐19, sepsis). (B) Within instrument scatterplots for both UniCel DxH 900 and CytoFLEX. (C) Between instrument scatterplots between for measurements of monocyte mean volume and variability. CytoFLEX measures refer to monocytes only (do not include double‐negative cells). For panel (A), there were no missing values for measures of monocyte variability (MDW and FSC‐SD), so for plots iii and iv, *n* = 52. There were two controls with missing MMV, so plots i and ii, *n* = 50. *p*‐values correspond to the test for association between paired samples (using Fisher *Z* transform). Panel (A) shows *n* = 50, as two controls had missing MMV values. *p*, *p*‐value; *r*, Pearson product–moment correlation coefficient

The distribution of monocyte parameters of mean volume (MMV and FSC‐mean) and volume variability (MDW and FSC‐SD) are summarized in Table 1 and Figure 2A, which showed that UniCel DxH 900 was able to detect differences between controls and disease that were not apparent in CytoFLEX; while CytoFLEX detected differences in volume variability between COVID‐19 and sepsis that were not identified using UniCel DxH 900.

### 3.2 Within and between‐instrument comparison of parameters

For both UniCel DxH 900 and CytoFLEX analysis, monocyte volume variability appeared to increase in concert with monocyte mean volume (Figure 2B). This relationship between volume variability and average volume (the within instrument comparison) followed a clear positive linear trend when both parameters were measured in UniCel DxH 900, but was less well defined for CytoFLEX measures.

**FIGURE 3:**
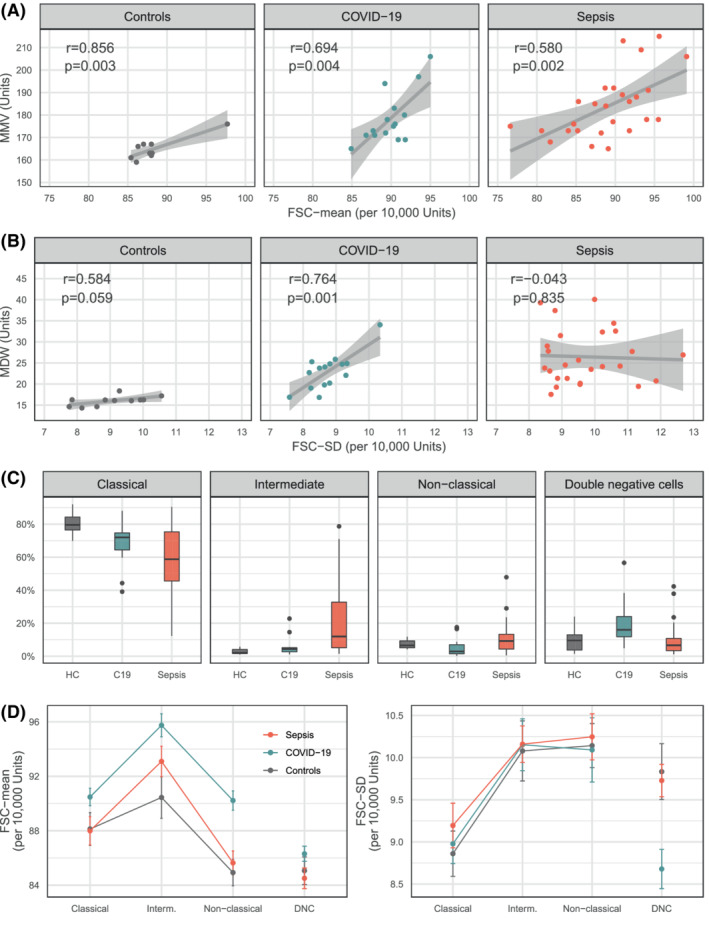
(A) Scatterplots between UniCel DxH 900 and CytoFLEX measurements of monocyte mean volume (stratified on patient‐group). (B) Scatterplots between UniCel DxH 900 and CytoFLEX for mean volume variability (stratified on patient‐group). (C) Boxplots displaying the proportion of cell subtypes identified in the CytoFLEX CD14 versus CD16 density plot (live, lineage‐negative cells) across patient‐groups. (D) CytoFLEX average cell volume (left) and average cell volume variability (right) across cell subtype and patient‐groups. *p*‐values correspond to the test for association between paired samples (using Fisher *Z* transform). In panels (A) and (B), correlation of MMV and FSC‐mean on controls shows *n* = 9, as two controls had missing MMV values. For panel (C), the denominator for the calculation of proportions was HLA‐DR^+^ cells, defined as blood mononuclear cells with HLA‐DR^+^ expression, and lack of B, T, NK markers. ‘HC’ is Healthy controls, and “C19” is COVID‐19. In panel (D), “Interm.” is Intermediate monocyte and ‘DNC’ is double‐negative cells. *p*, *p*‐value; *r*, Pearson product–moment correlation coefficient

The between‐instrument comparison is shown in Figure 2C, for measurements of monocyte mean volume (MMV vs. FSC‐mean in the left plot of Figure 2C) and mean volume variability (MDW vs. FSC‐SD in the right plot of Figure 2C). Overall, both measures of mean volume correlated moderately well (*r* = .543, *p* < .001), but measures of volume variability correlated poorly in the overall sample (*r* = .239, *p* = .088). However, when stratifying between‐instrument correlations according to sample type, all strata showed an increase in the magnitude of the coefficients except for measurements of variability in sepsis (Figure 3A shows the stratification of mean volume measurements and Figure 3B the stratification of variability measurements). For the sepsis group, correlations between MDW and FSC‐SD remained non‐significant (*r* = −.043, *p* = .835) even when accounting for malignancy, septic shock, bacteraemia, and SOFA score at admission.

The inclusion of double‐negative cells did not affect the correlations in the stratum of controls or sepsis. However, the between‐instrument correlation for COVID‐19 samples (both mean volume and variability) became weaker when double‐negative cells were included in the analysis suggesting (i) that double‐negative cells are unlikely to be included in the MMV and MDW parameters—if that were the case, we would have expected an increment in the strength of the between‐instrument correlation from inclusion of double‐negative cells in CytoFLEX parameters, and (ii) that double‐negative cells are particularly influential in the COVID‐19 stratum.

### 3.3 Monocyte subpopulation frequencies in COVID‐19 and sepsis

Using data from the Cytoflex analysis, we observed that the relative frequencies of monocyte subpopulations were different in controls, COVID‐19 and sepsis (Figure 3C). In both disease states, the proportion of classical monocytes (out of all HLA‐DR+ cells) dropped, but more so in sepsis than COVID‐19 (i.e. the median proportion was 58.8% in sepsis, 72.0% in COVID‐19, and 79.6% in healthy controls). In sepsis samples, this relative decrease in classical monocytes was matched by increases in the proportions of both intermediate (median 12.0%) and non‐classical cells (median 9.3%), whilst in COVID‐19 mostly the double‐negative population appeared to increase in frequency (median 16.0%).

### 3.4 Cell subpopulation phenotypic properties

Figure 3D shows the mean FSC‐mean (left) and FSC‐SD (right) with standard errors (SE) for each cell subtype and across the different patient‐groups. These show that different HLA‐DR+ cell subsets have characteristic volumetric properties, which are altered to different degrees in COVID‐19 and sepsis. Using FSC‐mean as a measure of cell volume, we first observed that intermediate monocytes were—on average—the largest monocyte subtype across all strata; furthermore, the ratio between the mean volume of classical and intermediate monocytes was higher in disease compared to controls, and constant in both paradigms of injury (i.e. intermediate monocytes were 5% larger than their corresponding classical monocytes, in COVID‐19 and sepsis; and only 2% larger than classical monocytes in healthy controls). Second, all COVID‐19 cell subtypes (including double‐negative cells) were on average, larger than their counterparts in the control group (Figure 3D, left); but in sepsis, only the intermediate monocytes were distinctly larger than the controls. Lastly, COVID‐19 non‐classical monocytes—although larger than all the other non‐classical monocytes—had the same mean FSC‐mean as COVID‐19 classical monocytes, whereas both sepsis and controls showed non‐classical monocytes being considerably smaller than classical cells.

The average volume variability for each monocyte subtype (estimated as the mean FSC‐SD) was very similar in all strata (Figure 3D, right). Overall volume variability was higher in intermediate and non‐classical cells compared to classical monocytes, but this could be an expression of higher inherent variability in the volume of CD16+ cells or a consequence of their lower relative counts. Notably, the volume variability of double‐negative cells was significantly lower in COVID‐19 compared to control and sepsis. Again, this could be an expression of volume homogeneity in this population or larger cell counts.

**FIGURE 4:**
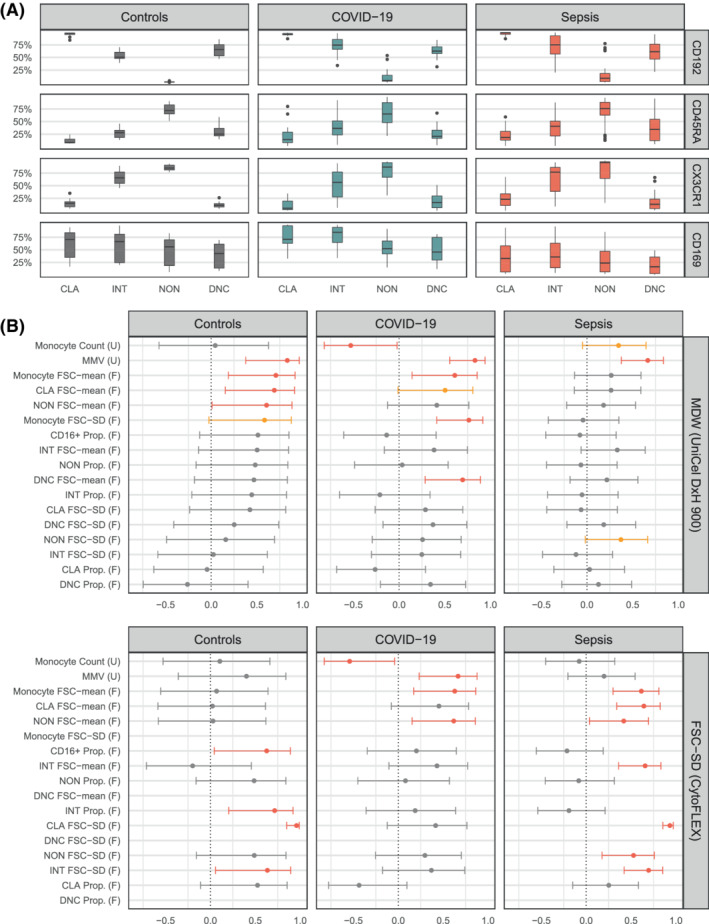
(A) Boxplots displaying the proportion of cell subtypes identified in the CytoFLEX CD14 versus CD16 density plot (live, lineage‐negative cells) expressing either CD192, CD45RA, CX_3_CR1 or CD169, across patient‐groups. (B) Forest plot displaying the correlation coefficient (and 95% CI) between (top) MDW and different monocyte parameters across patient‐groups, and (bottom) between FSC‐SD and different monocyte parameters across patient‐groups. (F) parameter obtained from CytoFLEX. (U) parameter obtained from UniCel DxH 900. Monocyte parameters obtained from CytoFLEX exclude double‐negative cells. Coefficient corresponds to Pearson product–moment correlation coefficient, and confidence intervals are given based on Fisher *Z* transform. *p* ≤ .050 are highlighted in red, *p* > .050 and *p* ≤ .090 for sepsis group are highlighted in orange. Correlation of MMV and FSC‐mean on controls shows *n* = 50, as two controls had missing MMV values. CD16^+^, CD16 positive cells, which include intermediate and non‐classical cells; CLA, classical monocytes; DNC, double‐negative cells; INT, intermediate monocytes; NON, non‐classical monocytes; Prop, proportion

When we investigated whether the changes in MDW correlated with changes in other phenotypic markers (CD192 [CCR2], CD45RA, CX3CR1, and CD169), we observed (Figure 4A) a remarkably consistent pattern of expression across HLA‐DR+ cells for all patient‐group strata. The only exception was CD169 which appeared upregulated in COVID‐19 and downregulated in sepsis.

### 3.5 Parameters correlated to volume variability (MDW and FSC‐SD)

When we compared the correlation between MDW and FSC‐SD against UniCel DxH 900 and CytoFLEX parameters (Figure 4B) we found (i) that the magnitude, direction, and significance of any given pair of correlates differed across sample groups suggesting a singular configuration of the monocyte population in each stratum, and (ii) that MDW and FSC‐SD shared correlates only in the COVID‐19 stratum. In this stratum, both measures of variability were positively correlated with monocyte volume (MMV had *r* = .831, *p* < .001 and FSC‐SD *r* = .666, *p* = .007) and negatively correlated with the UniCel DxH 900 monocyte count (*r* = −.529, *p* = .043 and *r* = −.542, *p* = .037 respectively).

We used multivariate analysis in an attempt to disentangle this complex interrelation of parameters and to compare the strongest predictors for both measures of monocyte variability in this study population. This multivariate analysis did not intend to explain why monocyte variability increases and had very specific exploratory objectives: (i) were the factors that most strongly predict overall FSC‐SD similar to those that predict MDW in this sample? (ii) did the double‐negative cell parameters affect MDW values? and (iii) could the variability in MDW (in this sample) be explained only using CytoFLEX variables. These results lack generalisability and are presented in the Data S1.

## 4 DISCUSSION

This was a descriptive analysis using 52 specimens that included healthy controls as well as two different paradigms of injury (COVID‐19 and sepsis). The aim was to compare monocyte volumetric parameters (of volume and variability) using two different instruments (UniCel DxH 900 and CytoFLEX) and to explore the phenotypic changes driving the increase of MDW in disease, leveraging the granularity included within flow cytometry data.

In this analysis, we observed moderate‐to‐good levels of between‐instrument correlations in control and COVID‐19 samples (for both volume and variability), but in sepsis, a correlation was only observed for measurements of volume (MMV vs. FSC‐mean). The univariate distribution of all volumetric parameters in sepsis showed the largest measures of dispersion (SD and IQR), despite having the highest number of samples, suggesting a high level of inherent variability within this subgroup. It is possible that the lack of correlation observed in sepsis was due to insufficient sample size for the level of variability in this subgroup.

In a homogeneous population, the variability of a given characteristic is inversely associated with the number of units and independent of the distribution mean (assuming random sampling and normality),^26^ but as flow cytometry research has shown, monocytes are far from homogeneous both in terms of function and volume.^14^, ^15^, ^16^ A closer inspection of the different monocyte subpopulations (CytoFLEX) revealed two fundamental points underpinning changes in monocyte volume heterogeneity. First, different monocyte subsets have a characteristic average volume in steady state (controls) with intermediate cells being the largest and non‐classical the smallest. And second, in disease each monocyte subset undergoes changes in volume and relative frequency to a different extent, depending on the model of injury.

So, in COVID‐19, all monocyte subtypes have larger volumes, with intermediate cells still being the largest, and non‐classical cells becoming so large that they are similar in volume to classical cells—which also become less prevalent. In other words, there is an increase in the overall average volume, but there is no (or little) increase in the range of volume across cells (compared to controls). In sepsis, instead, intermediate cells markedly increase in volume and proportion, extending the range of average volumes (from the largest intermediate to the smallest non‐classical), as classical cells (in the middle of the volume distribution) become less prevalent, the distribution of mean volume also becomes flatter.

This suggests that UniCel DxH 900 can detect differences between controls and disease that were not apparent with flow cytometry; while CytoFLEX detected differences in volume variability between COVID‐19 and sepsis that were not identified using UniCel DxH 900. As UniCel DxH 900 measures are based on two parameters only, it does not appear to be sensitive enough to capture differences between COVID‐19 and sepsis, only between healthy controls samples and samples from patients with either of the diseases studied.

In terms of limitations, this study was constrained by its non‐consecutive sampling methodology determined by the independent recruitment processes of the two different parent studies, hence neither demographic characteristics nor severity of disease are comparable across groups. The clinical variables available also depended on the parent study design and aims, so in many cases, it was not possible to quantify clinical differences across groups. Due to the nature of the research the number of available samples were small. This study was descriptive in nature and has not been powered to test any hypothesis, so random error cannot be ruled out. We have explored a large number of comparisons and type I error is likely to have arisen; therefore p‐values should be interpreted with caution. Furthermore, the observations in this descriptive study represent a snapshot of monocyte subpopulations at an unspecified time without being anchored to a known or estimated time of injury (time zero).

The blood monocyte pool is highly dynamic. In normal homeostasis, cells enter the blood after a short post‐mitotic dwell time in bone marrow (3–4 days). Even in the absence of an inflammatory stimulus, they exit the blood and are rapidly replaced (within a matter of days) by new cells emerging from bone marrow. In inflammatory states, these dynamics change. For example, following a single time‐point endotoxin challenge, all monocyte subsets rapidly disappear from the circulation. Classical cells are the first to be restored (at different rates from bone marrow and marginal pools in spleen and lungs), with intermediate and non‐classical cells following.^27^, ^28^, ^29^ Consequently, the proportion of monocyte subtypes at any given time will be a function of time from injury and the complex developmental trajectories and kinetic patterns that define the state of dynamic flux of monocyte subpopulations.^15^, ^16^ This current study strictly adhered to the international nomenclature to define human monocyte subsets.^18^ Nevertheless, with the advent of increased multiparameter flow cytometry, novel strategies are emerging to define monocytes.^30^ Furthermore, systemic inflammation can confuse the identification of leukocyte subsets where monocytes have exhibited CD56 membrane expression^31^ or a down regulation of HLA‐DR.^32^, ^33^, ^34^ By observing the international nomenclature, we were comparing the same cells as in healthy controls.

Given recruitment constraints, the time elapsed between admission and venepuncture varied between groups, being shorter for sepsis (IQR 1–3 days) than for COVID‐19 cases (IQR 17–34 days) limiting the generalisability of our findings, particularly with respect to disease states. However, the between‐instrument comparability remains valid, as sample pairs were taken simultaneously. Analysis using UniCel DxH 900 took place within 1 h of venepuncture, whilst PBMC processing (for CytoFLEX analysis) occurred within 12 h of venepuncture, with no significant differences in processing times between groups.

## 5 CONCLUSION

Having compared monocyte volumetric parameters retrieved by the UniCel DxH 900 analyser against the corresponding parameters measured using flow cytometry in conditions known to elevate MDW values (sepsis and COVID‐19), we observe that UniCel DxH 900 appeared able to detect differences between controls and disease that were not apparent with flow cytometry; while CytoFLEX detected differences in volume variability between COVID‐19 and sepsis that were not identified using UniCel DxH 900. This could be explained by both instruments being differentially sensitive at different ranges. As UniCel DxH 900 measures are based on two parameters only, it does not appear to be sensitive enough to capture differences between COVID‐19 and sepsis, only between healthy controls samples and samples from patients with either of the diseases studied. Flow cytometry (CytoFLEX) analysis captures a greater degree of detail and information about the changes in the configuration of the monocyte population that underpin the different immuno‐physiological processes characterizing each disease.

## FUNDING INFORMATION

This study was funded by Beckman Coulter, Brea, CA, USA. The funders had no role in the study design, data collection and analysis, results analysis and interpretation, decision to publish or preparation of the manuscript. The award was granted to D.M. The funder provided support in the form of salary support for M.C. and consumables for flow cytometry.

## CONFLICT OF INTEREST

The authors have no relevant financial or non‐financial interests to disclose.

## PATIENT AND PUBLIC INVOLVEMENT

The patients and the public were involved in the development of the study protocol.

## Supporting information


**Table S1**. Cell sorting and analysis antibodies.
**Table S2**. Beckman‐Coulter CytoFLEX S optical configuration and settings.
**Table S3**. Compensation matrix.
**Table S4**. Models characteristics.

## Data Availability

The data underlying this article are available in the article and in its online supplementary material.
