# Supplementary material for: Increased monocyte distribution width in COVID‐19 and sepsis arises from a complex interplay of altered monocyte cellular size and subset frequency
Source: Int J Lab Hematol. 2022 Aug 1:10.1111/ijlh.13941. Online ahead of print. doi: 10.1111/ijlh.13941 (PMC9538408; doi:10.1111/ijlh.13941)
Supplement: Supplementary file 1 — Table S1. Cell sorting and analysis antibodies. Table S2. Beckman‐Coulter CytoFLEX S optical configuration and settings. Table S3. Compensation matrix. Table S4. Models characteristics. [file IJLH-9999-0-s001.pdf]

METHODS (SUPPLEMENTARY)

Participants

The inclusion/exclusion criteria were different for each of the disease samples and were in line with those from the parent studies.

- For sepsis participants, the criteria were as follows: age (≥18 years), a high clinical suspicion of sepsis at ED admission, and a negative SARS-CoV-2 result confirmed by polymerase chain reaction (PCR).
- For COVID-19 participants, the criteria were slightly more restrictive. In addition to being required to have an age of ≥18 years, participants were required to have been admitted to a COVID-19 specific ward with SARS-CoV-2 infection confirmed by PCR and/or a clinico-radiological diagnosis of COVID-19. Samples from patients unlikely to survive over 28 days were excluded, as well as those who needed to be transferred to another hospital within 72 hours of admission, or those who had a history of immunosuppression (e.g. metastatic malignancy, congenital or acquired immunodeficiency, receipt of chronic systemic treatment with known immunosuppressant medications, or radiotherapy).

Processing samples (timelines)

For each participant, whole human peripheral blood was collected routinely in sterile EDTA vacutainer tubes (containing K2 dipotassium ethylenediaminetetraacetic acid) The date/time of venepuncture, as well as sample identifiers, were recorded and a unique study number was assigned. Samples were sent for FBC measurement following routine hospital procedures using haematology high-volume analysers (Beckman Coulter UniCel DxH 900). Once the FBC was processed, UniCel DxH 900 readouts and clinical data were obtained and anonymised. The excess material was collected and processed (within 12 hours of venepuncture) for the isolation of peripheral blood mononuclear cells (PBMCs) using the Ficoll-Paque procedure and subsequent flow cytometry analysis.

Ficoll-Paque procedure

Whole blood samples, diluted at a 1:1 ratio with phosphate-buffered saline (PBS), were layered – at room temperature – onto 5ml of Ficoll media solution. Thereafter, the samples were centrifuged at 400g for 20 minutes (at 20°C). PBMCs were then isolated by pipette, washed twice in warm RPMI 1640-medium (from Sigma-Aldrich) with 1% foetal calf serum (FCS), counted, and suspended in a freezing medium of FCS and 10% dimethyl sulfoxide (DMSO). Samples were stored at −80°C for at least 24 hours before being transferred to liquid nitrogen.

Direct labelling

Cryopreserved PBMCs were thawed rapidly, spun in RPMI 1640-medium with 1% FSC, washed, and resuspended in PBS. Thereafter, fluorophore-conjugated antibodies were used to label the target markers (direct staining). First, samples were diluted to 10 million cells/mL with PBS and incubated in the dark for 30 minutes at room temperature with 1uL of FVD520 (viability dye) conjugated with fluorescein isothiocyanate (FITC). Second, 5uL BioLegend Fc blocking agent was added and incubated for ten minutes at room temperature in the dark. This was done to block non-specific FcR-mediated staining without interfering with antibody-mediated specific labelling. Third, because multiple staining reagents conjugated with Brilliant Violet (BD) Horizon Brilliant fluorescent polymer dyes (Table 1s) were used, BD Horizon Brilliant Stain Buffer (50uL) was added to avoid fluorescent dye interactions causing staining artefacts and thus affecting data interpretation. After the addition of the brilliant stain buffer, all remaining antibodies were added together with 40uL PBS and incubated in the dark for 20 minutes at 4°C. After washing in PBS, cells were reconstituted in 250uL FACS fix buffer (2% formaldehyde in PBS), followed by incubation at 4°C in the dark for at least 30 minutes. The cells were spun and the FACS fix was replaced with 250uL FACS buffer before analysis. The antibody cocktail (Table 1s) was constituted using the dilutions provided in the manufacturer’s instructions.

Table 1s: Cell sorting and analysis antibodies.

Marker	Fluorochrome	Clone	Company	Reactivity	Cat no
Live/Dead*	FITC	FVD520	Invitrogen	--	65–0867–14
CD3	FITC	HIT3a	Biolegend	T lineage	300306
CD19	FITC	HIB19	Biolegend	B lineage	302206
CD20	FITC	2H7	Biolegend	B lineage	302304
CD56	FITC	5.1H11	Biolegend	NK cells	362546
CD66b	FITC	G10F5	Biolegend	Granulocytes	984102
CD45RA	PerCP-Cy5.5	HI100	Biolegend	Monocytes	304122
HLA-DR	BV605	G46-6	BD	Monocytes/DC	562845
CD14	APC-Cy7	6303	Biolegend	Monocytes/DC	367108
CD16	PE-Cy7	3G8	Biolegend	Monocytes/DC	302016
CX3CR1	APC	2A9-1	Biolegend	Monocytes/DC	341610
CD169	PE	7-239	Biolegend	Monocytes/DC	346004
CD192	BV421	K036C2	Biolegend	Monocytes/DC	357210

(*) cellular proteins (amines). Cat no: catalogue number.

Flow cytometry and gating strategy (details)

Optical configuration and settings are displayed in Table 2s. Hereunder, CytoFLEX will be used to refer to all flow cytometer measurements. Compensation was automatic using beads as controls, the matrix is shown in **Error! Reference source not found.**s. All data were exported using Flow Cytometry Standard (FCS) files, which were analysed using FlowJo software (FlowJo, LLC, version 10.6.2) and an eight-colour panel (details on staining antibodies are shown in Table 1s).

As the first step in our gating strategy, PBMC cell populations were highlighted using forward (FSC-H) and side-scatter (SSC-H) parameters to broadly identify monocyte populations based on their size and granularity (FSC/SSC properties). Following this, an FSC-H versus forward scatter area (FSC-A) density plot was used to gate singlets (so, only cells with approximate equal area and height were included). Once doublets were excluded, a panel of HLA-DR$^+$ versus live and lineage-negative cells was generated. CD3, CD19 and CD20 were used to eliminate T and B cells, CD56 to exclude natural killer (NK) cells, and CD66b to exclude granulocytes. The viability marker (to identify dead cells) was conjugated with the same fluorochrome as lineage-negative markers, so these two were indistinguishable. The result of this two-gate process was the identification of blood mononuclear cells with HLA-DR$^+$ expression, and lack of B, T, NK markers. This analysis will refer to this population as HLA-DR$^+$ cells.

Table 2s: Beckman-Coulter CytoFLEX S optical configuration and settings.

Laser	Detector name	Bandpass Filter	Fluorochromes
Blue	FITC	525/40	FITC,
(488nm)	PerCP	690/50	PerCPCy5.5
Yellow/Green	PE-TxRed	610/20	
(561nm)	PE	585/42	PE,
	PE-Cy5.5	690/50	
	PE-Cy7	780/60	PE-Cy7
Violet	PBlue	450/45	BV421
(405nm)	AmCyan	525/40	
	BV605	610/20	BV605
	BV650	660/10	
Red	APC	660/10	APC,
(633nm)	AF700	712/25	
	APC-Cy7	780/60	APC-Cy7,

Table 3s: Compensation matrix.

	FL1-A	FL2-A	FL3-A	FL5-A	FL6-A	FL8-A	FL10-A	FL13-A
FL1-A	1.000	0.033	0.000	0.000	0.000	0.004	0.000	0.000
FL2-A	0.000	1.000	0.021	0.159	0.000	0.000	0.000	0.105
FL3-A	0.000	0.025	1.000	0.192	0.000	0.000	0.000	0.046
FL5-A	0.000	0.002	0.042	1.000	0.000	0.000	0.000	0.227
FL6-A	0.000	0.000	0.000	0.000	1.000	0.006	0.000	0.000
FL8-A	0.000	0.081	0.002	0.001	0.039	1.000	0.167	0.044
FL10-A	0.007	0.149	0.000	0.000	0.000	0.032	1.000	0.016
FL13-A	0.001	0.010	0.000	0.084	0.000	0.000	0.008	1.000

FL1-A: dump FITC-A; FL2-A: CD45RA PerCP-A; FL3-A: CX3CR1 APC-A; FL5-A: CD14 APC-A750-A; FL6-A: CD192 PB450-A; FL8-A: HLA-DR Violet610-A; FL10-A: CD169 PE-A; FL13-A: CD16 PC7-A.

As the objective of the gating strategy was to isolate viable monocytes, an additional gate to identify cells expressing CD14 and CD16 was needed. Therefore, a two-parameter density plot was used, where CD14 was represented in the y-axis and CD16 in the x-axis. In said plot, the following subsets were identified: (i) classical monocytes defined as $CD14^{+}CD16^{-}$, (ii) intermediate monocytes as $CD14^{+}CD16^{+}$, (iii) non-classical monocytes as $CD14^{lo}CD16^{+}$, and (iv) double-negative cells as $CD14^{lo}CD16^{lo}$ [1]. In this analysis, all these subsets were considered separately, and double-negative cells were not included in the monocyte's measurements [2,3]. However, we conducted several sensitivity analyses for the correlation of MDW against FSC-SD (overall and across strata). In these analyses FSC measurements corresponded to monocytes only (main analysis excluding $CD14^{lo}CD16^{lo}$) and HLA-DR+ cells as defined above (i.e. monocytes plus $CD14^{lo}CD16^{lo}$).

Once the subsets were identified, the light scattering properties were extracted and single parameter histograms for CD192 (CCR2), CX3CR1, CD169 and CD45RA were generated to assess the subpopulation structure and function. CD192 was selected as mediates monocyte chemotaxis [4,5], CD45RA activation of peripheral monocytes [6,7], CX3CR1 recruitment [8,9]), and CD169 for its role in macrophage binding [10,11]. Fluorescence minus one (FMO) was used to establish population boundaries.

MULTIVARIATE ANALYSIS (SUPPLEMENTARY)

Two main predictive models were generated. Model A looked at monocyte CytoFLEX variables (FSC-SD, FSC-mean for classical, intermediate, and non-classical monocytes, and their relative frequencies) as possible predictors of FSC-SD. Model B, instead looked at the same monocyte CytoFLEX variables as possible predictors of MDW. Results are displayed in Table 4s. Since these are not causal models, the results will not explain why monocyte variability increases; but what factors (in this population) remained associated with volume variability when accounting for all the variables in the initial model. The following topics were inspected in this analysis: (i) which factors predict overall FSC-SD and which MDW in this sample, (ii) whether double-negative cell parameters affect MDW values, and (c) whether the variability in MDW can be explained only using CytoFLEX variables.

The final model A (n=52) had six predictor variables: monocyte FSC-mean, classical FSC-SD and non-classical cell proportion had a significant positive influence on monocytes FSC-SD; while classical FSC-mean, non-classical FSC-mean and proportion of intermediate cells had a significant negative influence on FSC-SD. Model A1 explored the same parameters but removed one potential influential point (n=51) identified when checking model assumptions. Although the model fit improved and residual error dropped (RSE), there was no change in the sign the final predictors, so this observation was kept in the model.

The final model B (n=52) had two predictor variables: intermediate FSC-mean had a positive influence on MDW, and classical proportion had a negative influence. Model B was built using only monocyte variables, i.e. double-negative parameters were not included. Model B1 used the same initial parameters as model B plus double-negative parameters. However, the final model selection was the same (using the same criterion and only changing the initial set of variables), indicating that double-negative cells do not have a significant influence on MDW, even when accounting for all CytoFLEX variables.

Model B was built using CytoFLEX parameters only, so two additional models were fitted: one using sample groups as a multiplicative parameter (model B2) and the second, using UniCel DxH 900 monocyte count and MMV (model B3). Model B2 seemed a sensible approach from a graphical and statistical point of view, but not from a functional point of view because UniCel DXH 900 does not use any information on diagnosis to calculate MDW. However, this model has less residual error (RSE) than model B, indicating that the terms for COVID-19 and sepsis contained additional information needed for the prediction of MDW.

Model B3, represents an improvement (compared to B and B2) in terms of fit and residual error, and like model B, continues to indicate that intermediate FSC-mean positively influenced MDW (in addition to MMV), and classical cells negatively influence MDW (FSC-mean instead of proportion). In this population, the model might not be able to establish if monocyte count is influencing MDW because there was no difference in this parameter between our COVID-19 and sepsis samples. Weak statistical evidence was noted against the hypothesis of no interaction ($p=0.094$) between MMV and FSC-mean of classical monocytes with a negative interaction. The residual error of the model with interaction B3i was slightly lower than that of model B3.

Hence, we find that although theoretically FSC-SD and MDW measure the same characteristic (variability of volume) monocyte factors associated with changes in their magnitude are not the same. The variability in MDW cannot be explained only using CytoFLEX variables.

Table 4s: Models characteristics

Model	Model Summary		Model predictors‡	Estimate (95% CI)
**A**	Outcome=	FSC-SD	Monocyte FSC-mean	0.392 (0.305, 0.479)
	n=	52	Classical FSC-SD	0.878 (0.780, 0.976)
	Adj-R^2=	0.904	Classical FSC-mean	-0.345 (-0.432, -0.259)
	RSE (σ)=	0.329, df 45	Non-classical FSC-mean	-0.050 (-0.079, -0.021)
	F-statistic=	80.80 (p<0.001)	Intermediate prop.	-0.010 (-0.018, -0.002)
			Non-classical prop	0.012 (0.000, 0.023)
**A1**	Outcome=	FSC-SD	Monocyte FSC-mean	0.259 (0.198, 0.320)
	n=	51*	Classical FSC-SD	0.914 (0.821, 1.007)
	Adj-R^2=	0.918	Classical FSC-mean	-0.229 (-0.283, -0.175)
	RSE (σ)=	0.305, df 45	Non-classical FSC-mean	-0.029 (-0.057, 0.000)
	F-statistic=	113.66 (p<0.001)	Non-classical prop	0.012 (0.001, 0.022)
**B**	Outcome=	MDW	Classical prop	-0.110 (-0.193, -0.027)
**B1**	n=	52	Intermediate FSC-mean	0.456 (0.145, 0.768)
	Adj-R^2=	0.204		
	RSE (σ)=	5.783, df 49		
	F-statistic=	7.52 (p=0.001)		
**B2**	Outcome=	MDW	Non-classical FSC-SD	1.243 (0.177, 2.310)
	n=	52	COVID-19	7.034 (3.098, 10.970)
	Adj-R^2=	0.421	Sepsis	10.295 (6.727, 13.863)
	RSE (σ)=	4.931, df 48		
	F-statistic=	13.37 (p<0.001)		
**B3**	Outcome=	MDW	Classical FSC-mean	-0.736 (-1.240, -0.231)
	n=	50**	Intermediate FSC-mean	0.450 (0.004, 0.896)
	Adj-R^2=	0.663	MMV	0.390 (0.303, 0.477)
	RSE (σ)=	3.72, df 46		
	F-statistic=	33.12 (p<0.001)		
**B3i**	Outcome=	MDW	Classical FSC-mean	2.538 (-1.422, 6.497)
	n=	50	Intermediate FSC-mean	0.458 (0.021, 0.896)
	Adj-R^2=	0.676	MMV	2.061 (0.054, 4.069)
	RSE (σ)=	3.65, df 45	Interaction term	-0.018 (-0.04, 0.004)
	F-statistic=	26.5 (p<0.001)		

Adj-R2: Adjusted R squared. RSE (σ): Residual standard error. df: degrees of freedom. p: p-value. p-values correspond to the F-test (variance-ratio test) for the final model with k-1 and n-k degrees of freedom (where k is the number of parameters including the intercept). Prop: Proportion.

‡ Model predictors list does not include intercepts. * Sensitivity analysis removing one control outside Cook's distance in Model A. ** Two controls had no MMV values. Model B and model B1 are shown together as both yielded the results using a different set of initial variables.

REFERENCES (SUPPLEMENTARY)

1. Ziegler-Heitbrock L, Ancuta P, Crowe S, et al. Nomenclature of monocytes and dendritic cells in blood. *Blood.* Oct 21 2010;116(16):e74-80. doi: 10.1182/blood-2010-02-258558.
2. Patel AA, Zhang Y, Fullerton JN, et al. The fate and lifespan of human monocyte subsets in steady state and systemic inflammation. *Journal of Experimental Medicine.* 2017;214(7):1913-1923. doi: 10.1084/jem.20170355.
3. Wong KL, Yeap WH, Tai JJ, Ong SM, Dang TM, Wong SC. The three human monocyte subsets: implications for health and disease. *Immunol Res.* Sep 2012;53(1-3):41-57. doi: 10.1007/s12026-012-8297-3.
4. Dutta P, Sager HB, Stengel KR, et al. Myocardial Infarction Activates CCR2(+) Hematopoietic Stem and Progenitor Cells. *Cell Stem Cell.* May 7 2015;16(5):477-487. doi: 10.1016/j.stem.2015.04.008.
5. Mack M, Cihak J, Simonis C, et al. Expression and characterization of the chemokine receptors CCR2 and CCR5 in mice. *J Immunol.* Apr 1 2001;166(7):4697-4704. doi: 10.4049/jimmunol.166.7.4697.
6. Clark EA, Ledbetter JA. Leukocyte cell surface enzymology: CD45 (LCA, T200) is a protein tyrosine phosphatase. *Immunology Today.* 1989/07/01/ 1989;10(7):225-228. doi: 10.1016/0167-5699(89)90257-0.
7. Nagasawa T, Kobayashi H, Aramaki M, Kiji M, Oda S, Izumi Y. Expression of CD14, CD16 and CD45RA on monocytes from periodontitis patients. *J Periodontal Res.* Feb 2004;39(1):72-78. doi: 10.1111/j.1600-0765.2004.00713.x.
8. Beziat V, Duffy D, Quoc SN, et al. CD56brightCD16+ NK cells: a functional intermediate stage of NK cell differentiation. *J Immunol.* Jun 15 2011;186(12):6753-6761. doi: 10.4049/jimmunol.1100330.
9. Nishimura M, Umehara H, Nakayama T, et al. Dual functions of fractalkine/CX3C ligand 1 in trafficking of perforin+/granzyme B+ cytotoxic effector lymphocytes that are defined by CX3CR1 expression. *J Immunol.* Jun 15 2002;168(12):6173-6180. doi: 10.4049/jimmunol.168.12.6173.
10. Hartnell A, Steel J, Turley H, Jones M, Jackson DG, Crocker PR. Characterization of human sialoadhesin, a sialic acid binding receptor expressed by resident and inflammatory macrophage populations. *Blood.* Jan 1 2001;97(1):288-296. doi: 10.1182/blood.v97.1.288.
11. Xiong YS, Zhou YH, Rong GH, et al. Siglec-1 on monocytes is a potential risk marker for monitoring disease severity in coronary artery disease. *Clin Biochem.* Jul 2009;42(10-11):1057-1063. doi: 10.1016/j.clinbiochem.2009.02.026.
